# Effects of sublethal and realistic concentrations of the commercial herbicide atrazine in Pacu (*Piaractus mesopotamicus*): Long-term exposure and recovery assays

**DOI:** 10.14202/vetworld.2020.147-159

**Published:** 2020-01-23

**Authors:** Mariana Cruz Delcorso, Paula Pereira de Paiva, Marcela Regina Paganuchi Grigoleto, Sônia C. N. Queiroz, Carla Beatriz Collares-Buzato, Sarah Arana

**Affiliations:** 1 Department of Biochemistry and Tissue Biology, University of Campinas, Campinas, SP, Brazil; 2 Laboratory of Residues and Contaminants, Embrapa Environment, Jaguariúna, SP, Brazil

**Keywords:** Brazilian ichthyofauna, genotoxicity, histopathology, liver and kidney lesions, recovery assay, ultrastructure

## Abstract

**Background and Aim:**

The commercial formulations of the herbicide atrazine (cATZ) are widely employed in Brazilian agriculture, and, as a consequence, ATZ has been found at levels above that established by law in the river basins in Brazil. Although the toxicity of ATZ in fish is well documented, there are few studies on the recovery capacity after cATZ exposure. This work aimed to evaluate, using several biomarkers, the toxic effects of long-term exposure to the sublethal (3.57 mg/L) and nonlethal realistic (3.00 µg/L) cATZ concentrations followed by a recovery assay, in fingerlings of a Brazilian teleost, the *Piaractus mesopotamicus* (pacu).

**Materials and Methods:**

Pacu fingerlings were housed in glass tanks and divided into the following experimental groups (two tanks/group): Exposure control = EC, recovery control = RC, the sublethal groups exposed to 3.57 mg/L of cATZ, (sublethal exposure group = SLE and sublethal recovery group = SLR) and the nonlethal groups treated with 3.00 µg/L of cATZ (nonlethal exposure group = NLE and nonlethal recovery group = NLR). The exposure assay was semi-static with a duration of 30 days and the recovery assay (after cATZ withdrawal) lasted 14 days. Several biomarkers were evaluated in fingerlings from all groups: The swimming behavior, the body weight gain, the micronucleus formation and nuclear alterations in erythrocytes, and the hepatic and renal histopathology analyzed by qualitative and semi-quantitative morphological methods (using light and electron microscopy).

**Results:**

No significant difference in weight gain was observed among the groups after the exposure and recovery assays. The sublethal exposure induced impaired swimming movements, significant histopathological alterations, including necrosis in the liver and kidney, and a significant increase in the frequency of micronuclei in erythrocytes. The nonlethal exposure induced only subtle histopathological changes in the liver and kidney. After recovery assay, no genotoxic alteration was noted in pacu exposed to sublethal concentration, while the cATZ-induced kidney damage was partially reversed but not the hepatic injury.

**Conclusion:**

cATZ exhibits long-term toxic effects on pacu, even at relatively low concentrations, affecting mainly the liver and the kidney, and the effects of sublethal concentration are only partially reversed after cATZ withdrawal.

## Introduction

Atrazine (ATZ) (2-chloro-4-ethylamino-6-isopropylamino-1, 3, 5-triazine), a s-triazine group of herbicide, is one of the most used herbicide worldwide, being largely employed in the cultivation of sugar cane, corn, and sorghum [1]. ATZ and its metabolites can persist in water and soil for decades, representing a potential long-term threat to the environment, since it has been regularly found in ground and drinking water, as well as in seawater [2]. ATZ and its metabolites have also been found in Brazilian rivers [3,4] and in drinking water sources in several Brazilian state capitals [5], usually below the concentration limit permitted by the Brazilian legislation (2.0 µg/L)[6]. However, levels above this limit, ranging from 7.0 µg/L to 28.3 µg/L of ATZ, have also been detected in water and sediments in Brazilian river basins [7,8].

The effects induced by ecologically relevant concentrations of ATZ in fish have been described in studies of qualitative meta-analysis [9]. Regarding the genotoxic potential of this herbicide, controversial results have been reported when comparing the standard ATZ (sATZ) and the commercial formulations (cATZ) [10-12]. Cavas [10] observed that, in acute genotoxicity assay, sATZ did not cause a significant increase in frequencies of micronucleus or nuclear alterations in *Carassius auratus* when compared to the control group, while cATZ induced a significant increase in frequencies of both markers of genotoxicity. In contrast, other authors have found a genotoxic potential for both sATZ and cATZ at sublethal concentrations in long-term assays [11,12]. However, these authors describe different results regarding DNA repair ability after exposure. Nwani *et al*. [11] observed a decrease in the frequency of genotoxic changes after long-term cATZ treatment, indicating a possibility of repair. In contrast, Zhu *et al*. [12] reported an increase in the frequency of genotoxic changes over time with sATZ. Since the presence of ATZ in water resources is due to the agricultural use of the commercial formulations, investigation of the genotoxicity and other biological effects of cATZ seem more relevant. Studies have also described hepatic and renal damage induced by ATZ [13-15], showing that these organs are important metabolizing sites of this herbicide [16-19]. Nevertheless, works on renal and hepatic recovery after long-term exposure to ATZ are scarce, being conducted mainly in common carp [13,16]. To the best of our knowledge, the long-term effect and recovery response after ATZ exposure have not been thoroughly investigated in another teleost, including species of the Brazilian ichthyofauna.

The present work aimed to investigate the pacu sensitivity and recovery ability after long-term exposure (30 days) to cATZ at a sublethal concentration (3.57 mg/L), determined in a previous ecotoxicological assay [15], and a nonlethal realistic concentration (3.0 µg/L). The teleost pacu, *Piaractus mesopotamicus*, was chosen because of its economic value for aquaculture and sport fishing in Brazil. In addition, pacu displays a wide geographic distribution, not only in Brazil but also in South America, which is one of the main criteria for choosing a good bioindicator of aquatic contamination and biomonitoring studies [20].Thus, for our study, several biomarkers were used, such as the swimming behavior, the body weight gain, the micronucleus formation and nuclear alterations in erythrocytes, and the hepatic and renal histopathology qualitative and semi-quantitative morphological methods (using light and electron microscopy).

## Materials and Methods

### Ethical approval

All experimental protocols used in this work were approved by the Ethics Committee on Animal Use (CEUA) of the University of Campinas (UNICAMP) under protocol # 2378-1/B.

### Fish maintenance

Pacu fingerlings (*P. mesopotamicus* – Holmberg 1887) were obtained from a fish farm located in Mogi Mirim city at São Paulo state (−22° 25’ 55’’ S and −46° 57’ 28’’ W). They were transferred to stock tanks, in the laboratory, containing dechlorinated and aerated tap water, where they were kept for 30 days for acclimation. The photoperiod was established as a 12/12 h light/dark cycle controlled by a timer. Fish were fed daily with a commercial fish feed containing 4-6-mm pellets (Pirá 36, Guabi Nutrição Animal – composition: Crude protein [min.] 36%, ethereal extract [min.] 8%, fibrous matter [max.] 6.5%, mineral matter [max.] 10%, calcium [max.] 1.6%, and phosphorus [min.] [0.8%]) at a rate of 1.5% of their body weight mean. Every 2 days, one-third of the volume of the water in the tanks was siphoned for the removal of the remains of organic matter and replacement with fresh water immediately.

Before each experiment, the fish were maintained for 7 days for acclimatization to the glass aquaria at similar conditions to those of the stock tank. During the acclimatization period, in stock tanks and glass aquaria, the water quality was daily evaluated pH value = 8.10±0.09 (QUIMIS 186, 400A model), water temperature = 20.88±0.14°C, toxic ammonia concentration = 0.014±0.005 ppm, and hardness bland. The two last parameters were evaluated using colorimetric kits (Kit Labcon test – Alcon^®^). The same parameters of the water quality were evaluated daily during exposure and recovery assays. Water parameters recorded during the exposure assay were: pH value = 8.19±0.12, temperature = 21.07±0.36°C, and toxic ammonia concentration = 0.011±0.008 ppm; and the parameters during the recovery assay were: pH value = 8.02±0.24, temperature = 20.71±0.56°C, and toxic ammonia concentration = 0.014±0.009 ppm.

### Chemicals

A stock solution of ATZ was prepared in distilled water from a concentrated suspension of cATZ (500 g/L) commercially available (Gesaprim 500 CIBA-GEIGY^®^, Syngenta Crop Protection LLC, Louisiana, USA), and all working solutions were made from this stock solution. The anesthetic 2-phenoxyethanol was purchased from Sigma (St. Louis, MO, USA), and the Embed 812 epoxy resin – Epon kit was purchased from EMS (Hatfield, PA, USA). All chemicals used were analytical grade.

### Analytical chemistry of the water sampling

To determine the concentration of ATZ in the water from the glass tanks during the sublethal and nonlethal exposure experiment, 100 mL of water were collected from the treated and control groups 24 h after the cATZ addition to the aquaria, and periodically after the replacement of ATZ concentration at the 3^rd^, 7^th^, 14^th^, and 30^th^ days as well as during the recovery period, at the 1^st^, 7^th^, and 14^th^ days.

Water samples were transported and kept under refrigeration until analysis at 4°C in the dark. Before the analysis, the samples were filtered using 0.45 µm Millex HN Syringe Filters with a Nylon Membrane. For the qualitative and quantitative determination of ATZ in the samples, a high-performance liquid chromatography system was used. The system used had an automatic injector (Sil-10A), a quaternary pump (CTO-10A), and a UV/visible detector (SPD-10AV). The chromatographic separation was performed using a reversed-phase Lichrosorb RP-18 Column (250×4.6 mm, particle size of 5 µm, 100 A, Phenomenex) and was carried out with isocratic elution with a mobile phase of acetonitrile/water (50:50, v/v) and flow rate of 0.6 mL/min. The detection was done at 220 nm, and the runtime was 15 min. Qualitative and quantitative determinations were carried out using an external standard. The analytical curves were constructed using an sATZ (Chem Service, 98.1% purity) diluted in Milli-Q water. When necessary, the samples were diluted by a factor to fit the working range of the standard curves or concentrated using a rotary evaporator. The limit of detection method was 1 µg/L, the limit of quantification was 3 µg/L, and the relative standard deviation was ≤16.3%.

### Sublethal and nonlethal exposure and recovery assays in pacu fingerlings

After acclimatization in the laboratory, the wet body weight of each fish was measured, given a total weight mean of 12.64±0.04 g, and, then the fingerlings were randomly distributed in glass tanks (dimensions: 45 cm×32 cm×35 cm, 50 effective liters) containing dechlorinated and aerated water. Two glass tanks were designed for each experimental group, namely, the control groups (exposure control = EC and recovery control = RC), the sublethal groups exposed to 3.57 mg/L of cATZ, (sublethal exposure group = SLE and sublethal recovery group = SLR), and the nonlethal groups treated with 3.00 µg/L of cATZ (nonlethal exposure group = NLE and nonlethal recovery group = NLR). The sublethal concentration used here corresponded to 1/8^th^ of the LC_50_ of ATZ in pacu (28.58 mg/L), previously determined by Peterlini [21] and the nonlethal concentration is a realistic concentration already found in Brazilian rivers [8].

Six fingerlings per glass tank were used and the exposure and recovery assays were repeated twice in independent experiments, totalizing 72 fish. The exposure assay was semi-static, with the replacement of ATZ solutions (or dechlorinated water in the case of the control groups) that occurred every 72 h in the proportion of one-third of the volume of the glass tanks during the whole assay period of 30 days. After the 30^th^ day, all fish from EC, SLE, and NLE groups were euthanized by deep anesthesia in a glass tank containing 2-phenoxyethanol (diluted 1:600 in dechlorinated water). After the exposure period, the renewal of the water from the recovery glass tanks was initiated (RC, SLR, and NLR) with the replacement of clean dechlorinated water at the same proportion and frequency used in the exposure assay for 14 days. On the 14^th^ day, all fish from the recovery groups were euthanized as the exposure group.

After euthanasia, the wet body weight was obtained (final wet weight), the blood smears were prepared and tissue fragments were collected for histopathology analysis from all specimens (n = 12 fish/group).

### Micronucleus test in erythrocytes

**Figure-1:**
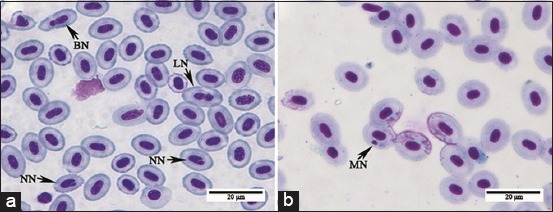
Micronuclei (MN) and nuclear abnormalities found in erythrocytes of *Piaractus mesopotamicus* (pacu) from control and commercial formulation of atrazine-exposed groups. Note the presence of lobed nuclei (LN), notched nuclei (NN), and blebbed nuclei (BN) indicated by arrows in (a) and MN in (b). 10% Giemsa solution.

The fish caudal fin was dissected to collect peripheral blood from a caudal vein using a heparinized microhematocrit capillary tube. Blood smears were air-dried overnight, fixed in pure methanol for 10 min and stained with 10% Giemsa solution for 10 min. Four slides were prepared for each fish, and 2000 erythrocytes were double-blind scored from each slide. Using a light microscope, the number of micronuclei (MN) and nuclear abnormalities (NAs), such as blebbed nuclei, lobed nuclei, and notched nuclei was registered in each pacu (Figure-1a and b) following the morphological criteria described by Carrasco *et al*. [22] The frequency of MN and NAs was calculated according to the following equation:


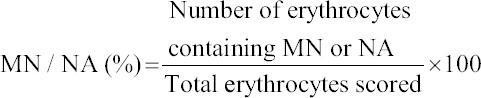


### Histopathological analysis by light microscopy

Representative samples from liver and kidney (trunk kidney) from each fish were fixed in *10*% neutral *buffered formalin* and Bouin’s solution, respectively, at room temperature for 24 h and were washed with tap water for 12 h. The samples were subsequently dehydrated using a graded series of ethanol, cleared in xylene, and embedded in paraffin. Sections (4 µm thick) obtained with an electronic microtome (Leica RM 2145) were stained with Hematoxylin-Eosin.

For inclusion in methacrylate (Leica Historesin^®^ Embedding Kit-7022 18 500), fragments of approximately 0.5 cm were fixed in calcium formaldehyde for 24 h. Tissue sections of 2 μm were stained with Hematoxylin-Floxin.

Stained sections were examined with a Nikon Eclipse E-800 light microscope (Nikon, Japan) equipped with a Cool SNAP-Pro color video camera (Media Cybernetics, San Diego, CA, USA), and images of the sections were captured using the Image Pro-Plus software (Media Cybernetics – version 4.1.1.2).

The degree of histopathological alterations of liver and kidney samples was evaluated and double-blind scored using the protocol described by Bernet *et al*. [23] Score values, ranging from 1 to 3, were given according to the importance represented by the histological lesion in terms of hepatic and renal functionality. The attribution of these scores was done by classifying the observed alterations according to the criteria described by Bernet *et al*. [23]. The alterations and their corresponding score values are given in Table-1.

**Table-1:** Histopathological alterations (biomarkers) and respective scores assessed in the liver and kidney of *Piaractus mesopotamicus* (pacu) exposed to the commercial formulation of atrazine.

Organ	Score	Alteration
Liver	1	Vacuolated cytoplasm
	1	Cellular hypertrophy
	1	Intracytoplasmatic deposits
	2	Vacuolated nuclei
	3	Focal necrosis
	1	Intercellular edema
	1	Vacuolated cytoplasm
Kidney	1	Granular degeneration
	2	Tubular hyperplasia
	3	Tubular necrosis

For the calculation of the degree of hepatic and renal damage for each animal, a modification of the method of Poleksic and Mitrovic-Tutundzic [24] was employed. The alteration severity index (I) was calculated according to the following equation, considering the score value (1, 2, or 3) given to each alteration as described above: I = Σ1+10 Σ2+100 Σ3. Once the index value (I) was obtained, the severity of the impairment of the organ was classified as follows: I of 0-10 = normal functioning; I of 11-20 = light to moderate damage; I of 21-50 = moderate to severe alterations; and I >100 = irreparable damage [24].

### Immunohistochemical analysis

Immunohistochemical analysis was performed to identify the preductular and ductular cells in the liver using the primary monoclonal antibody against cytokeratins AE1/AE3 (Dako Co. code M3515, dilution 1:50) and the EnVision Detection System kit (Dako Co. code K4065).

### Histopathological analysis by transmission electron microscopy

Liver and kidney samples were collected from four fish per group and fixed in modified Karnovsky solution (2.0% paraformaldehyde and 2.5% glutaraldehyde in 0.1 M phosphate buffer, pH 7.4) at 4°C for 24 h. Post-fixation was performed with 1% osmium for 1 h at 4°C. The material was washed with a glucose-saline solution, dehydrated in acetone and embedded in EMbed 812 epoxy resin. Uranyl acetate and 2% lead citrate were employed to contrast the ultrathin sections (60-70 nm) that were examined with a Zeiss LEO 906E Transmission Electron Microscope.

### Statistical analysis

Multiple comparative analyses of the data (expressed as means±SD or SEM) from the different experimental groups were performed using the analysis of variance followed by the Bonferroni *post* *hoc* test to compare pairs of means. The significance level was set at p<0.05. All statistical analyses were done using the GraphPad Prism Software version 5.00 (GraphPad Software, San Diego, CA, USA).

## Results

### cATZ concentration in experimental glass tanks

No trace of ATZ was detected in water samples from glass tanks of the control groups (EC and RC). In the aquaria of the SLE, during the 30 days of exposure assay, the concentration average of cATZ was 2.94±0.19 mg/L, which corresponded to 82.35% of the nominal concentration. The concentration of cATZ in the SLR aquaria during the 14 days of recovery assay was 0.399±0.003 mg/L, representing 11.17% of the sublethal nominal concentration. In the aquaria of the NLE group, during the period of exposure, the average concentration of cATZ was 4.00±0.05 µg/L, which was 33.33% above the nonlethal nominal concentration. cATZ concentration in water samples from the NLR tanks during recovery assay was below the detection limits.

### Mortality, swimming and feeding behavior observations, and weight gain evaluation

No mortality occurred in the control or treated groups during exposure and recovery assays.

Changes in feeding and swimming behavior were not observed in the control groups (EC and RC) and the groups exposed to nonlethal concentration (NLE and NLR). However, fish exposed to the sublethal concentration (SLE) showed a lower interest in searching for food (attested by the overplus of food often seen at the bottom of the aquaria) and presented darkening of the skin during the first 2 weeks of the exposure test. With the continuity of the exposure test, only a few specimens maintained this feeding behavior and also showed decreased swimming movements. During the recovery period (SLR group), a recovery of normal swimming behavior was noted but the darkening of the skin was maintained.

The comparative analyses of the wet body weight at the beginning of the exposure assay, after 30 days and at the end of the recovery assay (14^th^ day), indicated that there was no significant difference regarding this parameter among the groups (Table-2).

**Table-2:** Fish wet body weight before and after commercial formulation of atrazine exposure and recovery assays.

Experiment	Group	Initial wet weight	Final wet weight
Exposure	Control	12.87±2.59	15.58±2.43
	Sublethal	12.50±1.45	14.86±2.32
	Nonlethal	12.62±1.57	15.20±1.66
	Control	12.53±1.13	16.53±2.22
Recovery	Sublethal	12.68±1.39	16.03±1.76
	Nonlethal	12.66±1.48	15.92±2.02

Data expressed as means±SD. No significant difference was observed among the groups (One-way analysis of variance followed by Bonferroni’s post-test).

### Micronucleus test in pacu erythrocytes

The genotoxicity assay revealed a significant increase in the occurrence of MN and NAs in peripheral blood erythrocytes of pacu fingerlings from the SLE group compared to the other groups in the exposure assay (Table-3).

**Table-3:** Frequency (%) and total number of MNs and NAs observed in peripheral blood of pacu fingerlings after commercial formulation of atrazine exposure and recovery assays.

Experiment	Group	MN %	MN total	NA %	NA total
Exposure	Control	0.0021±0.0047	2	0.5344±0.4293	516
	Sublethal	0.0104±0.0112*	10	0.8208±0.3838	793
	Nonlethal	0.0031±0.0054	3	0.6052±0.3921	583
	Control	0.0000±0.0000	0	0.6073±0.04378	584
Recovery	Sublethal	0.0031±0.0054	3	0.7438±0.3812	714
	Nonlethal	0.0010±0.0035	1	0.6698±0.5095	646

(n=12 fingerlings/experimental group). (Total number of cells screened/group=96.000). Data expressed as means±SD.

* p<0.05 in comparison with control (One-way analysis of variance followed by Bonferroni’s post-test). MN=Micronucleus, NAs=Nuclear abnormalities

In the recovery assay, the comparative analysis did not indicate any significant difference among the groups concerning the occurrence of MN or NAs in pacu erythrocytes (Table-3).

### Histopathological and ultrastructural changes and severity index of organ damage

#### Liver

**Figure-2:**
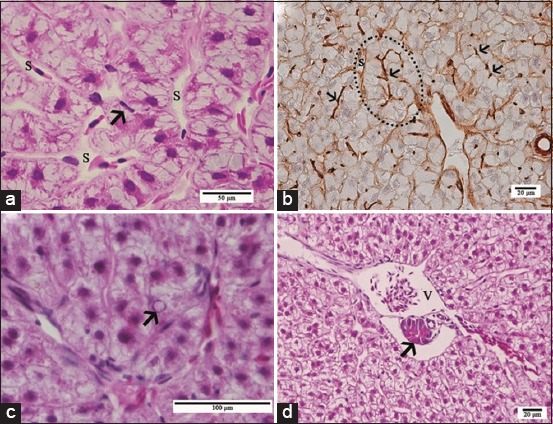
Light micrographs of control pacu liver. (a) Liver with a typical tubular arrangement, which consists of hepatocytes, with vacuolated cytoplasm, arranged in tubules, and displaying the apical surface facing the preductular epithelial cells (arrow) and the basal region directed to sinusoids (S). Historesin^®^, Hematoxylin-Floxin. (b) The preductular epithelial cells (arrows) in the center of the tubule lined by hepatocytes (demarked) and capillary sinusoid (S) near the basal region of the hepatocyte. AE1/AE3 cytokeratin antibody immunostaining. (c) Hepatocyte with nuclear vacuolization (arrow) H and E. (d) Note the intra-hepatic exocrine pancreas (arrow) in the adventitia of the portal vein branches (V). H and E.

In control and treated pacu fingerlings, the liver sections, as observed by light microscopy, presented the typical histological characteristics of a tubular arrangement, which consists of hepatocytes, with vacuolated cytoplasm, arranged in tubules with the apical region of the hepatocytes directed to the preductular epithelial cells (Figure-2a), immunodetected with the AE1/AE3 antibody (Figure-2b), and the basal region was facing the sinusoidal capillary (Figure-2a and b). Sparse hepatocytes with nuclear vacuolization were noted in the liver from all groups (Figure-2c).

Liver stroma in all groups also showed the typical histological characteristics described in fish. Furthermore, the intrahepatic exocrine pancreas was observed in the adventitia of the portal vein branches (Figure-2d). Light microscopy analysis showed no changes in structures of the biliary system or exocrine pancreas (data not shown) in fish from cATZ-exposed groups (SLE, SLR, NLE, and NLR).

**Figure-3:**
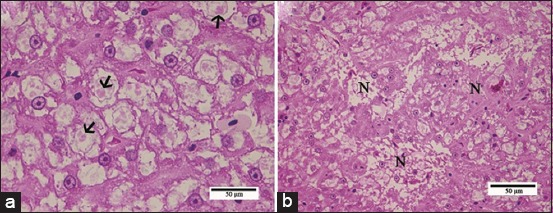
Light micrographs of pacu liver from sublethal recovery group. (a) Hypertrophic hepatocytes with large cytoplasmic inclusions containing globular or filamentous acidophilic material (arrows) (b) necrosis area (N). Historesin^®^, Hematoxylin-Floxin.

Liver of pacu from SLE and SLR groups presented several histopathological changes in the parenchyma that included hepatocyte hypertrophy and large cytoplasmic inclusions that were more frequently noted in hepatocytes from the SLR group, containing globular or filamentous acidophilic material (Figure-3a), as well as focal necrosis (Figure-3b). In fingerlings from the NLE and NLR groups, only hepatocellular hypertrophy, with the presence of inclusions as described above, was observed in the liver. The frequency of animals, from each experimental group, with different severity indexes of liver damage is shown in Table-4. Only fish exposed to sublethal cATZ concentration (SLE and SLR groups) showed severe liver damage in comparison with those of other groups. These analyses also indicated that there was no hepatic recovery from the exposure to sublethal cATZ concentration.

**Table-4:** Frequency (%) of the liver alteration severity index (I) after commercial formulation of atrazine exposure and recovery assays.

Experiment	Group	0-10	11-20	21-50	>100	I
Exposure	Control	100	-	-	-	2.25±0.43
	Sublethal	66.66	8.33	-	25	29.83±46.05*
	Nonlethal	66.66	33.33	-	-	5.83±4.91
	Control	100	-	-	-	2.17±0.37
Recovery	Sublethal	50	25	-	25	29.83±42.06*
	Nonlethal	75	25	-	-	4.74±4.21

I of 0-10=Normal functioning; I of 11-20=Light to moderate damage; I of 21-50=Moderate to severe alterations; I >100=Irreparable damage. n=12 fingerlings/experimental group. Data of I are expressed as means±SD.

* p<0.05 (One-way analysis of variance followed by Bonferroni’s post-test)

**Figure-4:**
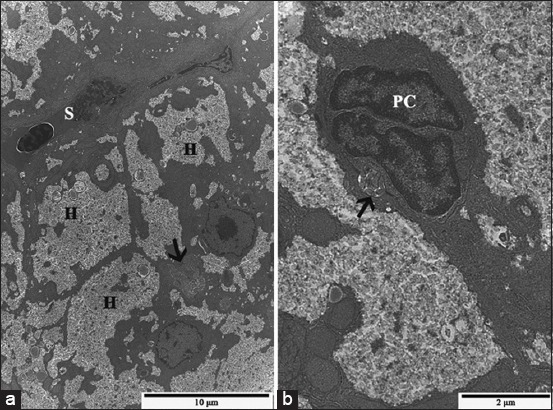
Transmission electron micrographs of control pacu liver. (a) A bile canaliculus filled with microvilli (black arrow), delimited by the plasma membrane of hepatocytes (H), and the basal surface of hepatocytes directed to sinusoids (S). (b) A canaliculus lumen with hepatocyte microvillus processes (black arrow) and the preductular epithelial cell.

**Figure-5:**
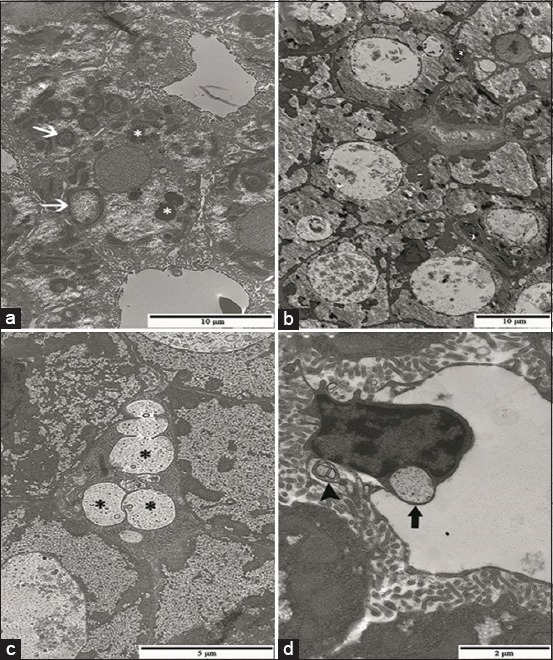
Transmission electron micrographs of liver from pacu exposed to commercial formulation of atrazine. (a) Note hepatocytes with altered mitochondrial shape (white arrow) and several lysosomes (*) in the sublethal exposure group. (b) Hepatocytes with large lysosomes and granular inclusions in fingerlings from the sublethal recovery group (SLR). (c) Preductular cells with residual bodies (*) in the SLR group (d) residual bodies in the endothelial cells of hepatic sinusoids (black arrow) and myelin figures in the perisinusoidal space (arrowhead) in the nonlethal exposure group.

The ultrastructure of hepatocyte and biliary system cells of fish from EC and RC groups display normal features usually described for the liver of teleost (Figure-4a and b). In the SLE group, the ultrastructural analysis revealed hepatocytes with altered mitochondrial shape and an increased number of lysosomes (Figure-5a). In addition to these ultrastructural changes, the SLR group presented hepatocytes (Figure-5b) and preductular cells (Figure-5c) with relatively large lysosomes containing a granular and lamellar material, considered as residual bodies, while the cellular junctions between these two cells were morphologically preserved. Ultrastructure of bile canaliculi, endothelial cells, and perisinusoidal space was preserved in both SLE and SLR groups. Meanwhile, the liver of pacu from the NLE group showed large residual bodies within hepatocytes and in the endothelial cells lining the sinusoids (Figure-5d), similar to those observed in the SLR group, and eventually, myelin figures in perisinusoidal space were also seen. The liver of fish from the NLR group displayed ultrastructure similar to that observed in control groups, except for the sparse mitochondria with altered shape seen in hepatocytes.

#### Kidney

**Figure-6:**
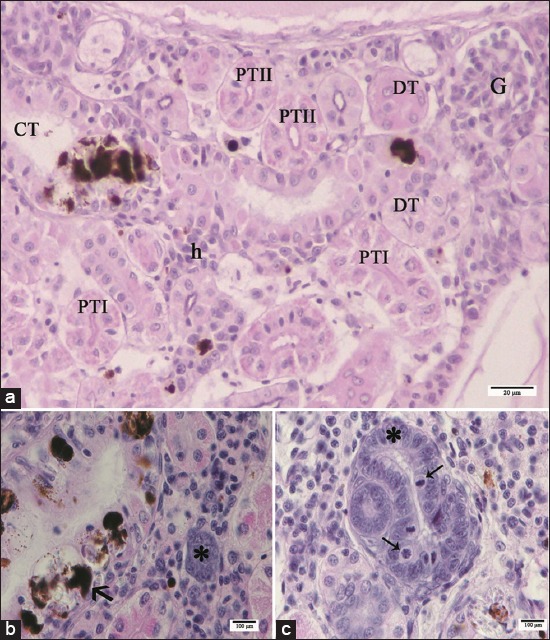
Light micrographs of control pacu kidney. (a) Note the glomerulus (G) and renal tubules surrounded by hematopoietic tissue (h). PTI: Proximal tubular segment I covered by columnar cells larger than of the cells of the PTII, note both tubules with prominent brush border. DT: Distal tubule characterized by cuboidal epithelial cells devoid of brush border. CT: Collecting tubule lined by pseudostratified epithelium. Note the presence of *Myxozoa* renal infection and infiltrate of melanomacrophages in the CT (arrow). (b) Nephrogenic response characterized by a basophilic mass of epithelial cells (*) and the presence of *Myxozoa* renal infection and infiltrate of melanomacrophages in the CT (arrow). (c) Note mitotic figures (arrows) in nephrogenic structures (*). H and E. PTII: Proximal tubular segment II.

**Figure-7:**
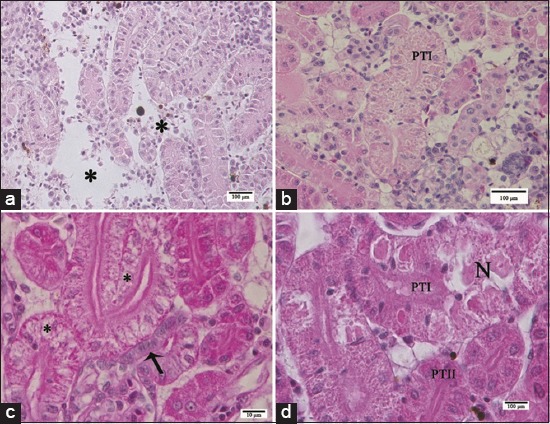
Light micrographs of pacu kidney from the groups treated with commercial formulation of atrazine. (a) Areas with edema (asterisks) in the nonlethal exposure group. (b) Observe a proximal tubule segment I (PTI) displaying granular and droplet degeneration. (c) Note cytoplasmic vacuolization (asterisks) in PTI cells. Isthmus (arrow). (d) PTI necrosis and proximal tubule segment II with no signs of damage. Photomicrographs in b, c, and d were obtained from pacu of the sublethal recovery group. H and E.

Trunk kidney of pacu from control groups (EC and RC) presented the usual morphology of glomerular kidney of teleost where the glomerulus (G) and renal tubules were surrounded by hematopoietic tissue (Figure-6a). Concerning the renal tubules, it was possible to identify the following segments: Isthmus, proximal tubule (Segments I and II [PTI, PTII]), distal tubule, and collecting tubule (CT) (Figure-6a). The isthmus (Figure-7c) was located between the G and the PT and consisted of a single layer of short cuboidal epithelial cells with basophilic staining. Both PTI and PTII are characterized by columnar cells with a prominent brush border, but the PTI cells were larger than PTII cells. The distal tubule (DT) is characterized by cuboidal epithelial cells devoid of brush border (Figure-6a). The CT (Figure-6a) lined by an epithelium that gradually changes from cuboidal to columnar or pseudostratified, with very eosinophilic cytoplasm and a relatively large lumen compared to the other segments; this tubule, in many portions, also presented some smooth muscle cells and connective tissue surrounding the basement membrane.

Light microscopy analysis also revealed *Myxozoa* renal infection in fish from all groups. *Myxobolus* spp. cysts were evidenced within the CTs, where infiltrates of melanomacrophages were commonly noted, or dispersed in the hematopoietic tissue (Figure-6a); meanwhile, the presence of the parasite was not observed in the other renal tubules or in the glomeruli. Areas with edema were also noted in all kidneys, including those from control groups. Necrosis of CT, due to the massive presence of the parasite, and melanomacrophages in this structure were seen in all experimental groups, therefore, these features were not considered for the score assignment of renal necrosis. Yet, the tubular necrosis reported here refers to that observed in the other tubular structures of the pacu kidney (i.e. PTI, PTII, and DT).

A nephrogenic response, characterized by a basophilic mass of epithelial cells, renal vesicles, and nascent nephrons (Figure-6b and c), was observed in pacu kidney from all groups and this feature was considered as a hyperplasia for score assignment and calculation of the severity index.

The trunk kidney from the SLE group exhibited areas with edema (Figure-7a) and with some PTI presenting granular and droplet degeneration surrounded by other renal tubules with no signs of damage (Figure-7b). Occasionally, cytoplasmic vacuolization was also noted in PTI cells (Figure-7c) and some fish displayed tubular necrosis (Figure-7d). In kidney from the SLR and NLE groups, tubular degeneration as described above was more frequently noted; edema was also observed in both groups, and eventually, cytoplasmic vacuolization and tubular necrosis were also seen. Pacu from the NLR group presented kidneys with a nephron structure very similar to fish from control groups.

Table-5 depicts the severity indexes of kidney damage of all experimental groups. The semi-quantitative analysis indicated that only the group SLE presented a significant increase in kidney injury in comparison with other groups of the exposure assay (EC and NLE). Meanwhile, there were no significant differences among the groups of the recovery assay.

**Table-5:** Frequency (%) of the kidney alteration severity index (I) after commercial formulation of atrazine exposure and recovery assays.

Experiment	Group	0-10	11-20	21-50	>100	I
Exposure	Control	75	25	-	-	10.25±0.43
	Sublethal	33.33	41.66	-	25	35.92±43.74*
	Nonlethal	8.33	75	-	16.66	27.42±35.89
	Control	91.66	8.33	-	-	9.25±2.80
Recovery	Sublethal	25	66.66	-	8.33	19.58±28.18
	Nonlethal	50	50	-	-	10.92±0.95

I of 0-10=Normal functioning; I of 11-20=Light to moderate damage; I of 21-50=Moderate to severe alterations; I >100=Irreparable damage. n=12 fingerlings/experimental group. Data of I are expressed as means±SD.

* p<0.05 (One-way analysis of variance followed by Bonferroni’s post-test).

**Figure-8:**
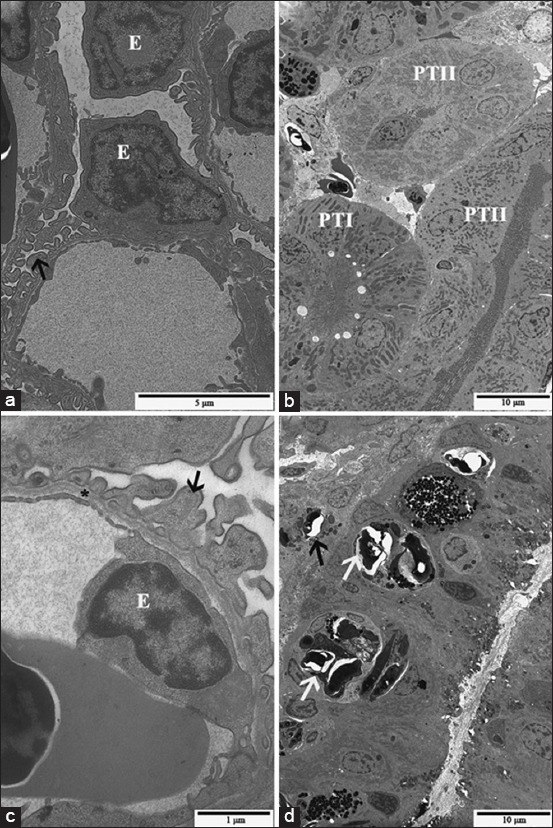
Transmission electron micrographs of pacu kidney from fish of the control group (a, b and d) and fish of the sublethal exposure group (c). (a) Glomerulus, where endothelial cells (E) and pedicels of podocytes (arrow) can be noted. (b) Observe the proximal tubule segment (PTI) I with the evident presence of vesicles and vacuoles associated with lysosomes and the apical region in comparison with the proximal tubule segment II (PTII). (c) Note endothelial cell (E), basal lamina (*) and pedicels of podocytes (arrow) with no morphological alterations. (d) *Myxobolus* spp. cysts in collecting tubules (black arrows) and in the hematopoietic tissue (white arrow).

Typical nephron ultrastructure of teleost was noted in trunk kidneys from EC, RC, and NLR groups (Figure-8a and b). The ultrastructure of PTI differed from that of PTII due to the evident presence of vesicles and vacuoles associated with lysosomes at the apical region and scarce mitochondria in the cytoplasm in the former (Figure-8b). Endothelial cells and podocytes of the glomerulus of fingerlings from cATZ-treated groups, in both exposure and recovery assays, presented the same characteristics observed in the control groups (Figure-8c). As described by light microscopy, the presence of *Myxobolus* spp. cysts in CTs (Figure-8d) and hematopoietic tissue was seen in pacu from all groups.

**Figure-9:**
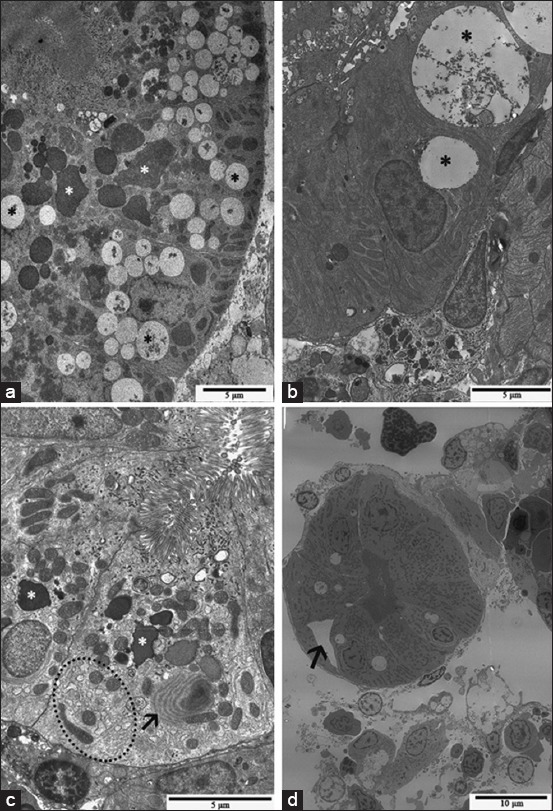
Transmission electron micrographs of the kidney of pacu from sublethal exposure group. (a) Observe proximal tubule segment I with cells containing several vacuoles, filled with a filamentous or globular material (black asterisks), and lysosomes (white asterisks). (b) Cell with large vacuoles (*) containing a filamentous material. (c) Cell with several lysosomes (white asterisks), myelin figures (arrow), and swollen smooth endoplasmic reticulum (demarcated area). (d) PTI with enlarged intercellular space (arrow).

In agreement with the histological data, the ultrastructural analysis of the cATZ-treated groups showed a significant impairment of PTI structure. Kidneys of pacu from SLE group presented PTI cells containing large vacuoles with a filamentous or globular material and several enlarged lysosomes (Figure-9a and b). Furthermore, some PTI displayed cellular degeneration, characterized by swollen cytoplasm as well as swollen smooth endoplasmic reticulum, and the presence of electron-dense inclusions and myelin figures (Figure-9c). In the kidney from the SLR group, similar ultrastructural changes were noted, except that enlarged intercellular space was eventually seen in PT in this experimental group (Figure-9d). Meanwhile, the kidney of some fish from the NLE group presented few ultrastructural modifications, similar to those described above for the PT tubules; but in general, a normal tissue organization was more often observed in this group.

## Discussion

In this study, we demonstrated that cATZ, at sublethal and nonlethal realistic concentrations, exhibits a long-term toxic effect on pacu, affecting several biomarkers (erythrocytes, liver, and kidney), that was partially reversible after the recovery period.

In pacu fingerlings, the cATZ genotoxic potential was only noted in erythrocytes, as assessed by the MN test, from fish exposed to the sublethal cATZ concentration (3.57 mg/L) that was fully reversed after the recovery assay. This result of cATZ exposure is in agreement with other works that showed DNA damage after long-term exposure to sublethal concentrations of cATZ [11] or sATZ [12] in other fish species. In contrast with our data, studies have demonstrated potential genotoxic effects of cATZ [10] and sATZ [25] in other fish at nonlethal concentrations in a short-term assay, however, these species were not challenged in a long-term assay, which would allow us to compare with our results. Indeed, few works investigated the recovery potential after ATZ long-term exposure, and as far as we know, the present work for the first time evaluated the genotoxicity induced by cATZ in pacu in both long-term exposure and recovery assays. Nwani *et al*. [11], studying the genotoxic potential of cATZ in erythrocytes and gill cells of *Channa punctatus* after exposure up to 35 days, have also described an increase in MN frequency, peaked on day 7 followed by a gradual non-linear decline afterward. They suggested that the activation of interdependent cascades of enzymes (i.e., superoxide dismutase and catalase), involved in attenuating the oxidative stress and repairing damaged DNA, was responsible for this decline in MN frequency. Genotoxic evaluation after recovery assay with other xenobiotics also showed a decline in the frequency of MN [26-28], which was explained in part by an increased splenic erythrophagia [26]. Therefore, it is plausible that both phenomena, activation of enzymes involved in cellular repair and an increased erythrophagia may also be accounted for the lack of detectable DNA damage in erythrocytes after exposure to the nonlethal concentration of cATZ and the recovery of this damage induced by sublethal cATZ concentration described herein.

A tubular-like arrangement of hepatocytes in pacu liver was demonstrated in the present study by light and electron microscopy and immunohistochemical stain methods, which is in agreement with the usual hepatic arrangement reported in teleosts by others [29,30]. Several hepatocellular changes were identified in pacu after long-term exposure to ATZ that included hypertrophy and the presence of large residual bodies and focal necrosis, with the necrosis being identified mainly in the liver from groups exposed to the sublethal ATZ concentration. These hepatocellular alterations were commonly associated with acute and chronic exposure to ATZ and other triazines in different fish species [31,32].

Residual bodies, such as those observed in pacu hepatocytes, probably result from the autophagy process that is a normal degenerative process in response to a variety of environmental stresses, such as xenobiotic exposure [33]. Residual bodies were also observed in preductular cells and endothelial cells of the hepatic sinusoids, indicating that cATZ exposure, at both sublethal and nonlethal concentrations, compromises the liver structure in general. Liver autophagy induced by ATZ was also described by Xing *et al*. [13] in common carp after exposure and recovery assays. Considering the ATZ sublethal treatment, it is noteworthy that the presence of residual bodies in the cytoplasm of pacu hepatocytes was more abundant in specimens from the recovery group than those from the exposure group. Similar results were described by Xing *et al*. [34], evaluating the ultrastructure of the brain of common carp exposed to ATZ, where autophagy was only noted in samples from the recovery group. These authors suggested that autophagy may play an important role in the cellular defense process after exposure to this herbicide. Indeed, recovery occurred in the liver of pacu after the withdrawal of the nonlethal cATZ exposure, suggesting that cellular strategies, such as autophagy, were effective in reversing the damage induced by this ATZ concentration. In contrast, no hepatic recovery was noted in fish exposed to the sublethal cATZ concentration. Importantly, all these observational results were semi-quantitatively confirmed by assessing the liver alteration severity index (I).

The liver plays a wide variety of important functions in teleosts, as in other vertebrates, such as protein synthesis, storage metabolites, bile secretion, detoxification [30], and participation in reproductive physiology, such as acting on vitellogenin synthesis [35]. Taking this into consideration, the hepatic impairment induced by ATZ in pacu, even at the nonlethal concentration, could result in marked physiological disturbances in this species. Therefore, the fact that this ATZ concentration is environmentally realistic, being already found not only in Brazilian rivers but also in river basins of other countries [36,37], raises the concern regarding the environmental impact of this herbicide to the ichthyofauna. Moreover, the frequent release of ATZ by agricultural activity and its long permanence in water and soil may be a hindrance to a successful recovery from the hepatic damage, as noted here.

Regarding the damage induced by cATZ to the pacu renal parenchyma, a large range of morphological alterations (from cytoplasmic vacuolization to marked tubular necrosis) was seen in the PT induced by the ATZ concentrations tested. In contrast, other tubules of the nephron displayed an apparent normal morphology. PT damage has been also reported by Oropesa *et al*. [38] that evaluated the effects of 45 µg/L of simazine for 14 days in carp. Our work represents one of the few studies that identified the tubule region damaged by xenobiotics; most of those studies do not describe in detail the tubular portions of the teleost nephron, which hinders the identification of the most affected nephron segment by ATZ exposure and other ATZs [14,39,40]. In mammals, the PT is the main target for several xenobiotics [41]. Few works have examined the specific location of enzymes involved in detoxification processes in renal tubules of teleost. Zodrow *et al*. [42] examined the acute toxicity of 2,3,7,8-tetrachlorodibenzo-p-dioxin (TCDD) in zebrafish and observed significant TCDD-dependent induction of CYP1A in PTs. It has been also suggested that PT is an important renal site of xenobiotic secretion in killifish since it expresses P-glycoprotein [43]. Based on these works and our morphological findings, we may suggest that the PT is the main site of ATZ action in the renal parenchyma of pacu.

In contrast with the PT segment, no changes were observed in the renal glomeruli structure, by light or electron transmission microscopy, even after pacu exposure to the sublethal cATZ concentration. Nevertheless, alterations to the renal glomeruli, which included the proliferation of podocytes and thickening of the glomerular basement membrane, have been reported in trout exposed for 28 days to different concentrations of this herbicide (5-80 µg/L) [44]. Yang *et al*. [39] also observed, in rare minnow exposed to different concentrations of ATZ for 28 days(0, 3, 10, 33,100 e 333 µg/L), a shrinkage of the glomerulus and increase in Bowman’s space at the concentration of 10 µg/L and no glomerular change with 3 µg/L of ATZ. The apparent discrepancy between our data and those described above may be explained by the use of different fish species and ATZ concentrations.

Kidneys from all pacu fingerlings sampled for histopathological analysis were infected by *Myxozoa*. This is a highly diverse taxon of endoparasites that are very often found in freshwater and saltwater fish and also in fish kept in aquaculture, which can cause damage to the tissue parasitized [45]. *Myxozoa* infection in pacu seems to cause several changes in CTs, including necrosis, but no effects in nephron tubules. Other studies have also described the infection of renal tubules by *Myxozoa* in pacu and another teleost [46,47], without specifying the renal segment as done here. In contrast, the *Myxozoa* infection appeared does not affect the renal tubules in juvenile cyprinids [48]. This difference in *Myxozoa* pathogenicity may be associated with the specific host-parasite interaction. The consideration of the presence of *Myxozoa* in the interpretation of the kidney damages is very important because the intercellular edema and tubular hyperplasia in pacu kidneys could be the result of a combined response to *Myxozoa* infection and ATZ exposure. The CTs necrosis and the PT necrosis could have been the stimulus to induce the nephrogenic response, characterized here by basophilic mass of epithelial cells and renal vesicles. Fish have a remarkable nephrogenic ability in comparison with mammals [49,50]. This fact should be taken into account when considering the degree of severity of kidney damage, evidenced by necrosis since the nephrogenic response can restore injured structures and enhance the degree of repair and the recovery of the organ. Accordingly, the pacu kidney presented a remarkable recovery from sublethal cATZ exposure, differing from the liver exposed to the same ATZ concentration, which showed no recovery during the period evaluated here.

Weight gain and other growth parameters have been considered important and sensitive parameters to assess the effects of xenobiotics since they represent an integration of a variety of physiological and environmental factors [51]. A decrease in weight gain and growth has been reported after ATZ and other triazine exposure [51-53]. Nevertheless, we observed no significant changes in pacu weight gain after long-term cATZ exposure, which agrees with other reports [54,55]. Despite the lack of body weight change, we observed a reduction in searching for food in the group exposed to sublethal concentration of cATZ during the first 2 weeks of exposure. Nieves-Puigdoller *et al*. [52] described a significant reduction in feed intake in Atlantic salmon after 10 days of exposure to 100 µg/L of ATZ. The adverse effects of ATZ on feeding behavior have been explained by an impairment of the olfactory epithelium affecting the chemoreception, as shown in salmonids [56], or as result from a decrease in locomotory ability [57]. In agreement with the latter idea, a reduction in swimming movements was observed herein in pacu after exposure to the sublethal cATZ concentration, and in other fish species after ATZ exposure, alone or in a mixture, at different concentrations [58,59].

## Conclusion

We demonstrated that long-term exposure to cATZ, at sublethal and nonlethal concentrations, had a genotoxic effect in erythrocytes and induced histopathological alterations of liver and kidney in a dose-dependent manner, which were partially reversible after herbicide withdrawal. Taken all together, our result indicated that pacu is a good bioindicator to freshwater contamination studies and that the liver is the most sensitive organ among those evaluated here, which emphasizes the inclusion of hepatic biomarkers in aquatic ecotoxicology assays using fish as a model. Furthermore, this work reinforces the importance of recovery assays in aquatic ecotoxicology for adequate evaluation of the impact of xenobiotics, particularly triazines, as well as to guide studies on bioremediation.

## Authors’ Contributions

SA designed the experiment. SA and CBC supervised the experiment, participated in the analysis and discussion of the data, drafted, and revised the manuscript. MCD participated in the experimental work and analysis of the data. PPP participated in the analysis and discussion of the data. MRPG collaborated with the experimental work. SCNQ performed the water analysis to evaluate the actual concentration of ATZ and collaborate in writing this part of the manuscript. All authors read and approved the final manuscript.
